# Effect of acupuncture on post-stroke dysphagia: a randomized controlled trial

**DOI:** 10.3389/fneur.2024.1391226

**Published:** 2024-06-21

**Authors:** Lin Bai, Hongliang Cheng, Peijia Hu, Qingqing Wang, Zhang Shilin, Zhiqiang Shen, Fangyuan Xu, Xingxing Su, Yiting Zhang

**Affiliations:** ^1^Department of Neurology, The Second Affiliated Hospital of Anhui University of Chinese Medicine, Hefei, China; ^2^The First Clinical Medical School, Anhui University of Chinese Medicine, Hefei, China; ^3^Fuyang City Sixth People's Hospital, Fuyang, China

**Keywords:** acupuncture, neurotransmitters, dysphagia, post-stroke, randomized controlled trial

## Abstract

**Introduction:**

Post-stroke dysphagia (PSD) is associated with various complications that increase morbidity and mortality rates. Acupuncture has been used extensively in China to treat these complications; however, its therapeutic efficacy remains uncertain. We therefore aimed to study the clinical effects of acupuncture on PSD.

**Methods:**

Patients (*n* = 101) were randomly divided into acupuncture (*n* = 50) and rehabilitation training control (*n* = 51) groups based on the treatment used. Both groups were treated once daily, 6 days a week, for a total of 4 weeks. Pulse oxygen saturation (SpO2) and standardized swallowing assessment (SSA) were performed before the intervention, 2 weeks into treatment, after the intervention (4 weeks post-intervention), and at a 6-month follow-up (28 weeks). The levels of hemoglobin (Hb) and albumin (ALB), and 5-hydroxytryptamine (5-HT) and dopamine (DA) were measured before the intervention, 2 weeks into treatment, and after the intervention (4 weeks), as nutrition and swallowing function indices, respectively.

**Results:**

Following the intervention, significant differences were observed between the acupuncture and control groups. The acupuncture group exhibited considerably superior enhancements in SpO2 and SSA scores at 4 weeks (*p* < 0.001). Moreover, this group demonstrated significantly greater improvements in Hb, ALB, 5-HT, and DA values 4 weeks post-treatment (*p* < 0.001). However, sex-based differences were not observed (*P* > 0.005).

**Conclusion:**

Acupuncture treatment can improve the swallowing function and nutritional status of patients with PSD, and increase the levels of 5-HT and DA. These findings strongly support the efficacy of acupuncture as a therapeutic intervention in patients with PSD.

**Clinicaltrial registration:** identifier, ChiCTR2100052201. (https://www.chictr.org.cn/).

## 1 Introduction

Stroke is the leading cause of death in China and ranks second globally. It is associated with significant morbidity and disability burden. Approximately 50% of patients affected by stroke experience dysphagia (1). Post-stroke dysphagia (PSD) is a complex sensorimotor syndrome, involving the disruption of the harmonious co-ordination of multiple muscle groups, the bulbar medulla, and cerebral cortex (2), which manifests when any component of the swallowing mechanism experiences dysfunction (3). It reduces the quality of life and increases the risk of malnutrition (4), electrolyte imbalance (5), aspiration (6), cough (7), and aspiration pneumonia (8). In the United States, adult patients with dysphagia experience an average increase of $6,243 in total hospital costs, and a 1.7-fold greater probability of in-hospital mortality than individuals unaffected by this condition (9). Despite the spontaneous recovery of swallowing in many patients with stroke, dysphagia persists in 11%–50% of cases at the 6-month mark. This condition is closely linked to heightened susceptibility to stroke-associated pneumonia (10), with a pneumonia incidence as high as 40% (11).

There are numerous autonomous predictors of PSD, including advanced age (12), higher National Institutes of Health Stroke Scale scores (13), greater modified Rankin Scale scores (14), lower Barthel Index scores (15), larger lesion volumes (16), cognitive impairment (17), and the presence of dysarthria (18), among others (19). The regions frequently implicated in dysphagia after a stroke encompass the subcortex, cortex, brainstem, corticobulbar tract, white matter, and other relevant areas. The brainstem and cerebellar structures play a crucial role in preserving the integrity of typical swallowing capabilities (20). Thus, the predictors of PSD and incidence of stroke may be closely related to the prognosis of PSD.

Numerous medical interventions are available for dysphagia, encompassing rehabilitative training, psychotherapeutic approaches, surgical interventions (21), neuromuscular electrical stimulation (22), pharyngeal electrical stimulation (23), and other modalities (24). Dietary changes, including alterations to the dimensions and consistency of the food bolus, as well as use of balloon dilatation and botulinum toxin injection, may be suitable in some cases (25). However, these procedures are associated with risks, including those of infection, bleeding, hoarseness, impairment of the recurrent laryngeal nerve, and even death (26).

Acupuncture has been proposed as an alternative approach with some evidence of efficacy (27). In previous studies, the use of acupuncture helped increase cerebral blood flow, facilitate augmented blood circulation within the affected regions of the tongue and pharynx, foster the establishment of cerebrovascular collateral circulation, and facilitate the restoration of the swallowing reflex (28). Moreover, the effect was better in pseudobulbar palsy than in bulbar palsy, and some evidence indicated that acupuncture could improve the nutritional status of patients presenting with dysphagia (29). This study therefore aimed to explore the application of acupuncture for the treatment of PSD.

## 2 Methods

### 2.1 Study design

This study is an assessor-participant-blinded, single-center, randomized controlled trial (RCT) conducted for patients diagnosed with PSD. The intervention period spanned 4 weeks, and the study was conducted between 27 October 2021 and 31 Dec 2022 at the Second Affiliated Hospital of Anhui University of Chinese Medicine. To ensure the integrity of the RCT, a proficient clinical trial specialist was engaged to oversee the process, providing valuable recommendations and making necessary amendments to address any project-related issues (30). The trial was recorded in the Chinese Clinical Trial Registry (reference number: ChiCTR2100052201) on 22 October 2021.

### 2.2 Participants

The study protocol was approved by the Second Affiliated Hospital of Anhui University of Chinese Medicine Research Board and Ethics Committee. The selected patients were outpatients and inpatients treated at this hospital between 27 October 2021 and 31 December 2022. All eligible patients provided informed consent prior to their participation. All patients met the diagnostic criteria for ischemic stroke (large-artery atherosclerosis type) (31) and pseudobulbar palsy (32).

The inclusion criteria were: Chinese ethnicity; an age of 50–80 years; meeting the diagnostic criteria for ischemic stroke and pseudobulbar palsy; stable vital signs and disease course within 90 days; clinical manifestations of dysphagia and cough caused by the sub-water test; physical examination revealing impaired movement of soft palate, throat, tongue, masseter and facial muscles, without atrophy or and tremor of tongue muscles; presence or hyperactivity of the soft palate reflex, and hyperactivity of the mandibular reflex is hyperactive; and willingness and ability to provide consent.

The exclusion criteria were: hemorrhagic stroke; bulbar palsy; pseudo-bulbar palsy for >90 days; conscious or cognitive functional impairment; serious diseases of the heart, liver, kidney, or endocrine system; multiple organ failure; and failure to complete acupuncture treatment within the scheduled time, or unwillingness/inability to provide consent.

### 2.3 Study procedures

**Figure 1:**
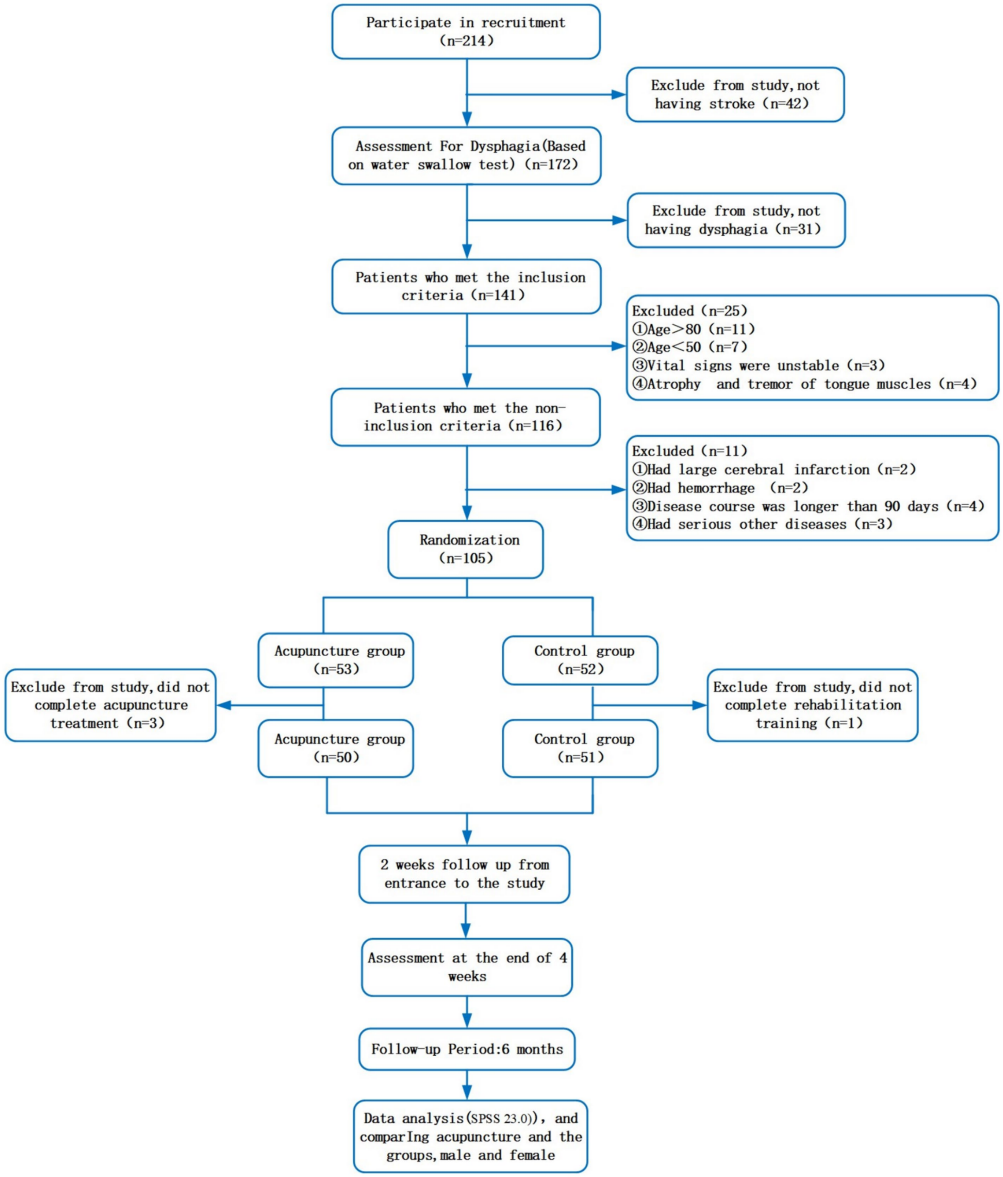
Flow chart of the trial.

The study population included 214 patients with PSD who were recruited at the study site (Figure 1). In total, 136 participants were enlisted, and their data were duly recorded in the Chinese Clinical Trial Registry. Regrettably, owing to the challenges encountered during the recruitment process, only 101 patients were eligible within the designated timeframe. Excel was used to generate a numbered randomization list, and patients were ordered based on their enrolment time. Random numbers were generated using the random number table method. Subsequently, patients who met the inclusion criteria were randomly allocated to either the acupuncture or rehabilitation training control groups. All healthcare personnel, excluding the acupuncturists, were unaware of the group assignment before each participant’s enrolment.

All adverse events that occurred during treatment were meticulously documented in case report forms. These adverse events included instances of fainting during acupuncture treatment, infections, pricking wounds, and various other complications.

### 2.4 Intervention

The baseline demographic and clinical characteristics of the trial groups are presented in Table 1. The male-to-female ratio in the acupuncture group was 19:31, while that in the control group it was 23:28. Patients received standard inpatient and outpatient care, as per the hospital protocol. In addition, patients in the acupuncture group underwent acupuncture administered by a highly experienced acupuncturist with 15 years of clinical experience and certification from the China Association of Acupuncture and Moxibustion.

**Table 1:** Demographic and clinical characteristics of the samples.

Variables	Acupuncture group	Control group	*p*-value
	(*n* = 51)	(*n* = 51)	
Age (years)	63.24 ± 7.33	63.90 ± 6.04	0.622
Sex (male/female)	19/31	23/28	0.546
Weight (kg)	73.86 ± 10.43	76.35 ± 7.64	0.175
Hypertension	24(48)	28(54.9)	0.493
Diabetes	23(46)	22(43.1)	0.775
Stroke course (days)	71.92 ± 2.34	72.39 ± 6.18	0.614
Case source			0.621
Outpatient	23(46)	26(51)	
Inpatient	27(54)	25(49)	
Marital status			0.451
Never married	3(6)	1(2)	
Married	42(84)	43(84.3)	
Divorced	1(2)	2(3.9)	
Widowed	4(8)	5(9.8)	
Educational level			0.67
Illiterate	6(12)	5(9.8)	
Elementary Guidance	19(38)	17(33.3)	
High school Diploma	20(40)	25(49)	
University	5(10)	4(7.8)	
Cigarette use	28(56)	26(51)	0.617
Alcohol use	20(40)	17(33.3)	0.492
Acupuncture experience	29(58)	34(66.7)	0.374

Data are presented as mean ± SD or number (%) unless otherwise stated.

#### 2.4.1 Acupuncture group

**Figure 2:**
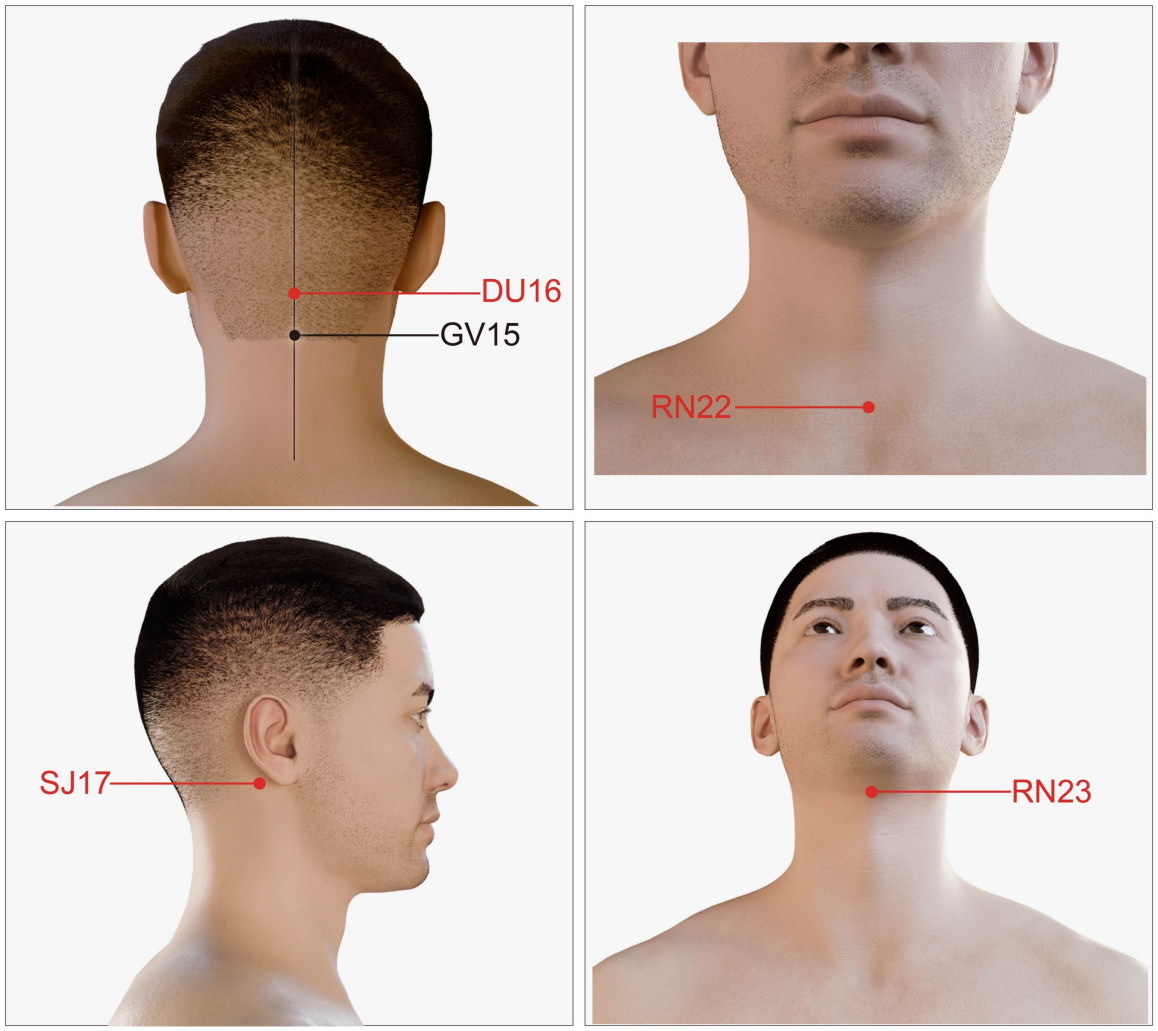
Location of the acupoints used in the study.

The acupuncture group was treated using “Tongdu Tiaoshen” acupuncture (28). The key points utilized for all patients in the acupuncture group were RN22 (Tiantu), DU16 (Fengfu), SJ17 (Yifeng), and RN23 (Lianquan) (Figure 2). When selecting the RN22 point, the patients were in the supine position without any pillows, and their heads were rotated to the right. When inserting the needles into the superior fossae, a vertical penetration of 10–15 mm was performed along the chest, followed by a subsequent adjustment of the needle tips in a downward direction. Subsequently, the needles were advanced 75–125 mm along the inside of the sternal handles, resulting in patients experiencing the sensation of chest distension. The needles were then twisted to induce chest tightness. When selecting the DU16 acupoints, the patients were positioned in the prone position, with the hairline at the back of their necks raised by 25 mm. The acupuncturists proceeded to insert 0.38 × 50 mm needles in the direction of injection, targeting the prominentia laryngea at a depth of 25–40 mm; needle insertion was ceased upon the patients’ experience of acid swelling or electric shock sensations. When targeting the SJ17 acupoints, the acupuncturists employed 0.38 × 50 mm needles inserted in the direction of injection at a depth of 25–40 mm from the roots of the tongues, utilizing a needle manipulation technique involving clockwise rotation; needle insertion was ceased upon the occurrence of patient-reported swelling or swallowing sensations in the throat. When targeting the RN23 acupoints, the acupuncturists employed 0.30 × 40 mm needles inserted 30 mm into the direction of injection at the root of the tongue. Needle insertion was discontinued if patients experienced acidic swelling extending to the roots of their tongue. All the needles were incubated for 30 min. Acupuncture treatment was administered to the patients six times per week over 4 weeks.

#### 2.4.2 Control group

The following rehabilitation training therapies were used: cold stimulation—pharyngeal cold stimulation was administered by applying frozen cotton swabs to the palatine arches and pharyngeal regions, and patients were instructed to perform gentle swallowing exercises twice daily; swallowing on empty stomach—patients engaged in a 20-min period of swallowing prior to each of their three daily meals; tongue movements—patients’ tongues were stretched in all directions twice a day, with the tongue depressors used to provide some resistance; lip movements—patients opened their mouths, smiled, and blew for 5–10 s twice a day; cough and feeding training—patients consumed liquid or soft-textured food at a gradual pace, and underwent rehabilitation training therapy six times per week for 4 weeks.

### 2.5 Outcome measures

The primary objective of this study was to assess swallowing function using pulse oxygen saturation (SpO2) and standardized swallowing assessment (SSA) scores. Secondary objectives included the nutritive indices of hemoglobin (Hb) and albumin (ALB) levels; additionally, 5-hydroxytryptamine (5-HT) and dopamine (DA) levels were evaluated.

#### 2.5.1 SpO2 and SSA

SpO2 is an essential indicator of respiration and oxygen saturation in the human body. Previous studies have shown that pulse oximetry has the potential to accurately distinguish among individuals with and without dysphagia (33). We evaluated the decrease in SpO2 before and after the sub-water experiment using a fingertip pulse oximeter.

The SSA is a common method used to evaluate swallowing function. The SSA comprises three parts: Part 1, including consciousness, breathing, closure of the lips, soft palate movement, spontaneous cough, pharyngeal reflex, and head and trunk control; Part 2, allowing the patient to swallow 5 mL of water three times and observing the laryngeal movement, laryngeal function after swallowing, wheezing during swallowing, and whether there is repeated swallowing; and Part 3, asking the patient to swallow 60 mL of water and observing the swallowing condition for any abnormality. This scale can effectively evaluate swallowing function and aspiration in patients. The highest and lowest scores were 46 and 17 points, respectively; the lower the score, the better the swallowing function.

SpO2 and SSA were measured before the intervention, 2 weeks into treatment, after the intervention (4 weeks), and after the 6-month follow-up period (28 weeks).

#### 2.5.2 Hb and ALB

Hb and ALB levels were measured before the intervention, 2 weeks into treatment, and after the intervention (4 weeks). Venous blood was drawn after a period of fasting using a 2 mL EDTA anticoagulant tube and a 5 mL ordinary biochemical tube. The blood in the EDTA anticoagulation tube was analyzed via an automatic blood cell analyzer to measure Hb content using a colorimetric method. Centrifugation was then used to separate serum and plasma in ordinary biochemical tubes, and serum ALB levels were determined using biuret colorimetry.

#### 2.5.3 5-HT and DA

Previous studies reported that neurotransmitters are involved in swallowing (34). Monoamine neurotransmitters are central neurotransmitters including 5-HT, DA, and norepinephrine. It was demonstrated that 5-HT has a significant excitatory effect on both reflex and automatic swallowing (35), and DA agonists stimulate DA receptors in neurones to enhance swallowing function (36). Thus, this trial used 5-HT and DA as the laboratory index.

The levels of 5-HT and DA were measured before the intervention, 2 weeks into treatment, and after the intervention (4 weeks). Venous blood (5 mL) was collected after fasting, placed on ice, and centrifuged for 30 min. The serum extracted after centrifugation was stored at a low temperature away from light. Liquid–liquid extraction was performed to maintain a stable composition, and the contents of 5-HT and DA were determined by high performance liquid chromatography.

### 2.6 Data analysis

The sample size was calculated based on previous studies (28), considering an effect size of 0.86 and a power of 82% (α = 0.05, β = 0.1). After accounting for 20% attrition and 1:1 allocation, a sample size of 136 patients was calculated, with 68 patients were randomized to the acupuncture and control groups.

Data were analyzed using Statistical Package for Social Sciences (SPSS) software version 23 (SPSS Inc., Chicago, IL, United States). Quantitative variables were expressed as mean ± standard deviation. The Shapiro–Wilk test was used to check whether the variable had a consistently normal distribution; if it exhibited a normal distribution, a paired-sample t-test was used for intragroup comparisons, while an independent-samples t-test was used for comparisons between groups. Otherwise, the Mann–Whitney U test was used to compare the clinical features of the two groups, and qualitative variables are described counts numbers and percentages. *p*-values of <0.05 indicated statistical significance.

## 3 Results

In total, 214 participants were enrolled in the study between 27 October 2021 and 31 December 2022. Based on multiple screening criteria, a cohort of 105 patients was selected and subsequently allocated to two groups through randomization (acupuncture, *n* = 53; control, *n* = 52). Three and one participant from the acupuncture and control groups withdrew from the study due to noncompliance. Thus, a total of 101 subjects (acupuncture, *n* = 50; control, *n* = 51)—including 59 females (58.42%) and 42 males (41.58%)—aged 63.57 ± 6.69 years completed the study (Figure 1). There were no significant between-group differences in clinical features or other parameters, including age, sex, weight, hypertension, diabetes, educational level, cigarette use, and alcohol use (*p* > 0.05; Table 1).

### 3.1 SpO2 and SSA scores

**Figure 3:**
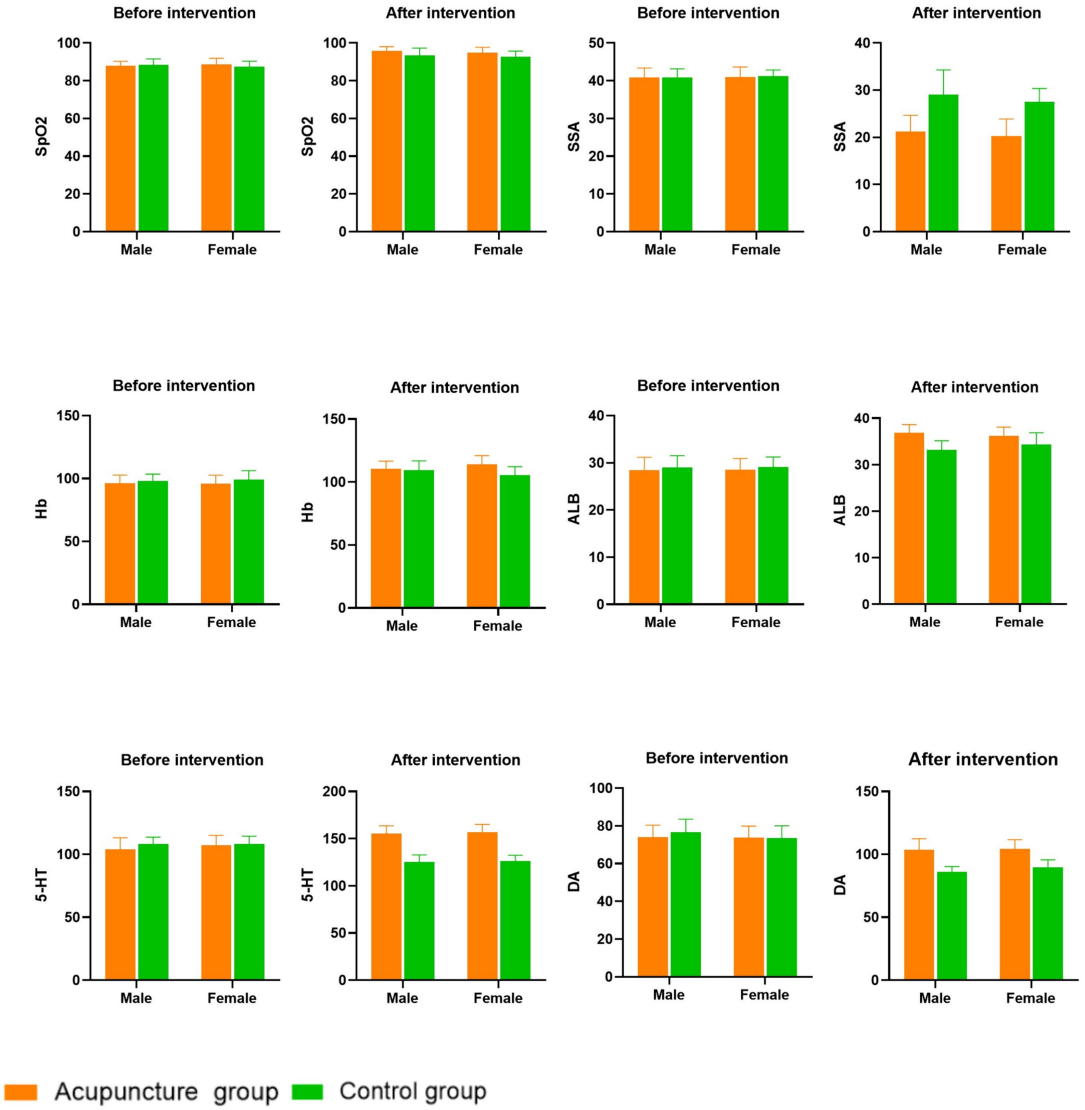
Comparisons of clinical evaluation indicators before and after treatment of two groups.

Following the intervention, participants in the acupuncture group exhibited significantly greater increases in SpO2 scores compared to those in the control group (Figure 3). Additionally, significant improvements in the SSA scores were observed in the acupuncture compared with control group (both *p* < 0.001; Table 2; Figure 3). Sex-based differences/effects were not observed for either outcome (*p* = 0.164 and *p* = 0.108, respectively; Table 2; Figure 3).

**Table 2:** Differences in SpO2 and SSA scores across study time points.

Variables	Sex	Acupuncture group	Control group	*p*-value/Sex	*p*-value/Group
		Mean ± SD	Mean ± SD		
**SpO2 score**					
Before intervention	Male	88.00 ± 2.29 (19)	88.43 ± 3.13 (23)	0.782	0.462
	Female	88.71 ± 3.13 (31)	87.39 ± 2.99 (28)		
	Total	88.44 ± 2.84 (50)	87.86 ± 3.07 (51)		
2-week treatment	Male	94.26 ± 2.40 (19)	91.30 ± 3.38 (23)	0.232	0.004
	Female	93.26 ± 2.78 (31)	92.86 ± 2.56 (28)		
	Total	93.64 ± 2.66 (50)	92.16 ± 3.03 (51)		
After intervention	Male	95.84 ± 2.19 (19)	93.48 ± 3.78 (23)	0.164	<0.001
	Female	94.87 ± 2.90 (31)	92.75 ± 2.94 (28)		
	Total	95.24 ± 2.61 (50)	93.08 ± 3.33 (51)		
6-month follow-up	Male	95.95 ± 1.81 (19)	92.70 ± 3.11 (23)	0.365	<0.001
	Female	96.03 ± 2.09 (31)	93.50 ± 2.44 (28)		
	Total	96.00 ± 1.97 (50)	93.14 ± 2.76 (51)		
**SSA score**					
Before intervention	Male	44.84 ± 2.52 (19)	40.91 ± 2.23 (23)	0.65	0.789
	Female	41.00 ± 2.65 (31)	41.18 ± 1.68 (28)		
	Total	40.94 ± 2.57 (50)	41.06 ± 1.93 (51)		
2-week treatment	Male	32.05 ± 2.37 (19)	35.43 ± 3.73 (23)	0.792	<0.001
	Female	32.48 ± 2.78 (31)	35.36 ± 3.94 (28)		
	Total	32.32 ± 2.61 (50)	35.39 ± 3.20 (51)		
After intervention	Male	21.26 ± 3.41 (19)	29.01 ± 5.24 (23)	0.108	<0.001
	Female	20.26 ± 3.61 (31)	27.54 ± 2.82 (28)		
	Total	20.64 ± 3.54 (50)	28.22 ± 4.11 (51)		
6-month follow-up	Male	17.68 ± 2.69 (19)	24.91 ± 2.57 (23)	0.466	<0.001
	Female	17.51 ± 2.26 (31)	25.82 ± 2.54 (28)		
	Total	17.58 ± 2.41 (50)	25.41 ± 2.57 (51)		

SpO2, Saturation of pulse oximetry; SSA, standardized swallowing assessment; 2-week treatment, 2 weeks following start of treatment.

### 3.2 Hb and ALB

There were no significant between-group differences in Hb or ALB levels before the intervention (*p* = 0.072 and *p* = 0.28, respectively). Participants in both the acupuncture and control groups had significantly higher contents of Hb and ALB at 2 weeks into the treatment period (*p* = 0.007 and *p* = 0.002, respectively) and after the intervention period (both *p* < 0.005; Table 3; Figure 3), compared with those before treatment. The improvements in Hb and ALB concentrations were more obvious in the acupuncture group, and the longer the acupuncture treatment time, the more obvious the increase in Hb and ALB concentrations. Sex-based differences/effects were not observed for either the Hb or ALB levels (*p* = 0.969 and *p* = 0.588, respectively; Table 3; Figure 3).

**Table 3:** Differences in Hb and ALB across study time points.

Variables	Sex	Acupuncture group	Control group	*p*-value/Sex	*p*-value/Group
		Mean ± SD	Mean ± SD		
**Hb(g/L)**					
Before intervention	Male	96.26 ± 6.51 (19)	98.00 ± 5.53 (23)	0.801	0.072
	Female	95.90 ± 6.76 (31)	99.04 ± 7.24 (28)		
	Total	95.04 ± 6.61 (50)	98.57 ± 6.48 (51)		
2-week treatment	Male	106.05 ± 4.92 (19)	100.83 ± 4.62 (23)	0.709	0.007
	Female	104.55 ± 6.14 (31)	103.21 ± 6.85 (28)		
	Total	105.12 ± 5.70 (50)	102.14 ± 6.02 (51)		
After intervention	Male	110.26 ± 6.34 (19)	109.73 ± 6.91 (23)	0.969	0.001
	Female	114.06 ± 6.86 (31)	109.95 ± 8.05 (28)		
	Total	112.62 ± 6.86 (50)	109.86 ± 7.58 (51)		
**ALB(g/L)**					
Before intervention	Male	28.47 ± 2.72 (19)	29.04 ± 2.48 (23)	0.89	0.28
	Female	28.58 ± 2.35 (31)	29.07 ± 2.19 (28)		
	Total	28.54 ± 2.47 (50)	29.06 ± 2.30 (51)		
2-week treatment	Male	32.68 ± 1.57 (19)	31.22 ± 1.54 (23)	0.665	0.002
	Female	32.19 ± 1.9 (31)	31.39 ± 1.91 (28)		
	Total	32.38 ± 1.83 (50)	31.31 ± 1.74 (51)		
After intervention	Male	36.84 ± 1.77 (19)	33.22 ± 1.93 (23)	0.588	<0.001
	Female	36.16 ± 1.91 (31)	34.36 ± 2.50 (28)		
	Total	36.42 ± 1.87 (50)	33.84 ± 2.46 (51)		

Hb, Hemoglobin; ALB, Albumin; 2-week treatment, 2 weeks following start of treatment.

### 3.3 5-HT and DA

There were no statistically significant differences in the levels of 5-HT and DA between the two study groups prior to the intervention (*p* = 0.053 and *p* = 0.377, respectively). Two weeks into the treatment period and after the intervention, it was observed that the serum levels of 5-HT and DA in both the acupuncture and control groups had increased. Notably, the acupuncture group exhibited significantly greater increases in these levels than the control group (all *p* < 0.001; Table 4; Figure 3). Sex-based differences/effects were not observed for either the 5-HT or DA levels (*p* = 0.471 and *p* = 0.14, respectively; Table 4; Figure 3).

**Table 4:** Differences in 5-HT and DA across study time points.

Variables	Sex	Acupuncture group	Control group	*p-*value/Sex	*p*-value/Group
		Mean ± SD	Mean ± SD		
**5-HT(ng/L)**					
Before intervention	Male	104.16 ± 9.06 (19)	108.48 ± 5.19 (23)	0.335	0.053
	Female	107.03 ± 8.08 (31)	108.43 ± 6.05 (28)		
	Total	105.94 ± 8.49 (50)	108.45 ± 5.62 (51)		
2-week treatment	Male	132.89 ± 5.52 (19)	115.57 ± 7.11 (23)	0.338	<0.001
	Female	132.23 ± 6.81 (31)	118.82 ± 6.70 (28)		
	Total	132.48 ± 6.30 (50)	117.35 ± 7.01 (51)		
After intervention	Male	155.63 ± 7.91 (19)	125.35 ± 7.32 (23)	0.471	<0.001
	Female	156.90 ± 8.22 (31)	126.25 ± 6.08 (28)		
	Total	156.42 ± 8.04 (50)	125.84 ± 6.62 (51)		
**DA(ng/L)**					
Before intervention	Male	73.95 ± 6.43 (19)	76.57 ± 6.97 (23)	0.213	0.377
	Female	73.74 ± 6.15 (31)	73.46 ± 6.54 (28)		
	Total	73.82 ± 6.20 (50)	74.86 ± 6.85 (51)		
2-week treatment	Male	95.16 ± 5.81 (19)	83.48 ± 6.87 (23)	0.656	<0.001
	Female	95.26 ± 9.55 (31)	84.68 ± 4.85 (28)		
	Total	95.22 ± 8.26 (50)	84.14 ± 5.82 (51)		
After intervention	Male	103.63 ± 8.80 (19)	86.17 ± 4.12 (23)	0.14	<0.001
	Female	104.32 ± 7.35 (31)	89.54 ± 6.11 (28)		
	Total	104.06 ± 7.85 (50)	88.02 ± 5.52 (51)		

5-HT, 5-hydroxytryptamine; DA, Dopamine; 2-week treatment, 2 weeks following start of treatment.

## 4 Discussion

This RCT demonstrated the effects of acupuncture on improving swallowing function and monoamine neurotransmitter levels. Our findings indicate that acupuncture treatment for PSD yielded positive outcomes, as evidenced by the significant improvement in SpO2 levels and SSA scores following a 4-week acupuncture therapy regimen. SpO2, which represents the oxygen concentration in the blood, is a significant physiological parameter in the context of respiratory circulation (37). Aspiration and coughing in patients with PSD lead to a decrease in SpO2. Our study revealed that patients who received acupuncture treatments for a duration of 4 weeks experienced a significant improvement in SpO2 scores compared with the control group. The SSA is an effective tool for the early identification of dysphagia; herein, we used it as a criterion for determining whether to remove the nasogastric tube. The acupuncture treatments improved the SSA scores of the acupuncture group, compared to those of the control group, from the treatment initiation to the end of the follow-up period.

Studies have shown that patients with anemia and malnutrition after PSD have a relatively poor prognosis (38), and low Hb levels are closely associated with PSD (39). Since the clinical experiment by Seltzer et al. (40), serum ALB has been widely used as a marker of nutritional status. Although the role of serum ALB in the assessment of nutritional status has been recently challenged, studies have shown that patients with low serum ALB levels are at risk of malnutrition (41). Our study revealed that the improvement in Hb and ALB concentrations was more obvious in the acupuncture than in control group after 2 weeks of treatment, suggesting some effects of acupuncture, which may be stronger if the intervention is administered earlier (42).

Monoamine neurotransmitters are central neurotransmitters, including catecholamines, indoleamines, DA, norepinephrine, and epinephrine (43); indoleamines are primarily 5-HT. Swallowing has been closely associated with neurotransmitters (34). The neurotransmitter 5-HT and its various mimetics have been found to have significant excitatory effects on both reflex and automatic swallowing. This effect is primarily observed in the lower brainstem, specifically in the nucleus tractus solitarii. The subnucleus centralis within the nucleus tractus solitarii is believed to be associated with a compact group of neurones that form the esophageal pattern generator circuitry (35), and these neurones project ventrally to the oesophagomotor portion of the nucleus ambiguous (44). The latest research shows that acupuncture, as a peripheral stimulation strategy, could directly improve the swallowing function in PSD model mice through the activation of motor cortex inputs to the nucleus tractus solitarii (NTS) and parabrachial nucleus (PBN). This is the M1-PBN-NTS neural circuit (45). DA agonists directly stimulate DA receptors within the neurones, leading to enhanced swallowing function; notably, aporphine and rotigotine—among other DA agonists—have been found to alleviate dysphagia and enhance involuntary pharyngeal function in individuals with Parkinson’s disease (36). In the semi-intact head preparation, 5-HT induces swallow-like movements, DA induces bite-like movements, and the regulation of feeding function by both DA and 5-HT is crucial, as they each contribute to organizing various aspects of feeding in distinct ways (46).

In traditional Chinese medicine, PSD is referred to as “throat impediment preventing speech,” while physiologically it is a form of brain damage resulting in throat function defects and swallowing disorders. These clinical manifestations suggest that the pharynx is closely related to the brain. Some authors consider the brain—pharynx—viscera pathway as a distinct system. The 12 meridians flow smoothly and converge in the pharynx, and the eight odd meridians are directly or indirectly connected with the pharynx. DU16 (Fengfu) in the Du pulse, SJ17 (Yifeng) in Shaoyang Sanjiao meridian, and RN23 (Lianquan) in the incumbent pulse, which all go through the throat. The meridians theory holds that wherever the meridians pass through, the disease of the place can be treated. Ren pulse is called the “sea of Yin pulse” and Du pulse is called the “sea of Yang pulse.” Ren pulse and Du pulse, which are important meridians in the treatment of PSD, can balance Yin and Yang and regulate qi and blood (47).

To our knowledge, this is the first RCT to study the efficacy of acupuncture on the monoamine neurotransmitters of PSD. The results of our study demonstrate that following 4 weeks of acupuncture, the serum 5-HT and DA levels increased in the acupuncture compared with those in the control group. However, this increase in DA level in both groups after the intervention were not significant when compared with 2 weeks into treatment. These findings are consistent with those of previous studies that demonstrated a strong correlation between the complex sensorimotor neural circuits involved in swallowing, and the neurotransmitter 5-HT. Deficiency of 5-HT leads to a decreased rate of licking and swallowing, exerting an excitatory effect on oropharyngeal swallowing. Animal experiments have demonstrated that a deficiency in 5-HT affects all three stages of swallowing, reducing licking and swallow rates, and increasing esophageal transit times. Consequently, acupuncture could directly improve swallowing function by promoting 5-HT production, while early supplementation of 5-HT may potentially mitigate or postpone the onset of dysphagia symptoms in individuals with neurologic diseases (48). However, pharmacological treatments affecting 5-HT levels are not currently being tested in trials aimed at improving swallowing function, and their use may have limitations, such as dysphagia associated with amyotrophic lateral sclerosis and Parkinson’s disease. Acupuncture treatment of dysphagia addresses the presenting symptoms and has a low risk of side effects, which are considered minor.

No previous study has examined the role of other monoamine neurotransmitters (such as noradrenaline and epinephrine) in dysphagia, and further studies are required to elucidate these relationships. Herein, acupuncture affected both subjective and objective outcome measures. However, the types of dysphagia vary, and the outcome of rehabilitation depends on the patient’s cognitive, motor, and sensory abilities (49), and the effects of acupuncture may not be satisfactory for patients with true bulbar paralysis (50).

## 5 Limitations

This study had several limitations; first, the duration of acupuncture treatment was only 4 weeks. Considering patients’ individual tolerance toward acupuncture therapy, this short duration of treatment may impact the recuperation of patients’ swallowing function. Additionally, the follow-up time may have been too short to observe the recovery of patients’ swallowing function more accurately. Second, the small sample size was another limitation of this study; although we recruited 210 patients, only 101 patients ultimately finished the trial. In the future, we aim to increase the sample size in a validation study to produce more robust results. Third, although we compared the efficacy of acupuncture in the study groups, we did not control for the site of stroke onset; this may affect the evaluation of the outcome. In the future we will incorporate more objective criteria and count the sites of cerebral vascular blockages to determine whether the effect of acupuncture on PSD is related to the site of vascular obstruction.

## 6 Conclusion

In conclusion, a 4-week acupuncture treatment improved swallowing function and the nutritional state of patients with PSD. Additionally, we found that acupuncture increased the levels of monoamine neurotransmitters (5-HT and DA). This suggests that monoamine neurotransmitters are involved in the functional regulation of target and effector organs. Moreover, acupuncture is easy to administer and can be used in clinical management for PSD.

## Data availability statement

The raw data supporting the conclusions of this article will be made available by the authors, without undue reservation.

## Ethics statement

The studies involving humans were approved by the Ethics Committee of the Second Affiliated Hospital of Anhui University of Chinese Medicine on 22 September 2021, 2021-zj-40, Hefei, China. The studies were conducted in accordance with the local legislation and institutional requirements. Written informed consent for participation in this study was provided by the participants’ legal guardians/next of kin. Written informed consent was obtained from the individual(s) for the publication of any potentially identifiable images or data included in this article.

## Author contributions

LB: Writing – original draft. HC: Writing – review & editing, Supervision, Project administration, Funding acquisition. PH: Writing – review & editing, Validation. QW: Writing – review & editing, Software, Data curation. ZShi: Writing – review & editing, Software, Data curation. ZShe: Writing – review & editing, Methodology. FX: Writing – review & editing, Visualization. XS: Writing – review & editing, Validation. YZ: Writing – review & editing, Validation.
